# Associations of Serological Biomarkers of sICAM-1, IL-1*β*, MIF, and su-PAR with 3-Month Mortality in Acute Exacerbation of Idiopathic Pulmonary Fibrosis

**DOI:** 10.1155/2020/4534272

**Published:** 2020-07-06

**Authors:** Xuran Li, Ying Zhou, Ruyi Zou, Haoran Chen, Xiaoqin Liu, Xiaohua Qiu, Yonglong Xiao, Hourong Cai, Jinghong Dai

**Affiliations:** Department of Pulmonary and Critical Care Medicine, Nanjing Drum Tower Hospital, Clinical College of Nanjing Medical School, Nanjing, Jiangsu, China

## Abstract

**Objective:**

To investigate prognostic values of serum biomarkers of soluble intercellular adhesion molecule 1 (sICAM-1), macrophage migration inhibitor factor (MIF), interleukin 1*β* (IL-1*β*), and soluble urokinase plasminogen activator receptor (su-PAR) in patients with acute exacerbation of idiopathic pulmonary fibrosis (AE-IPF).

**Methods:**

From August 2017 to November 2019, 122 consecutive IPF patients treated in our center were classified as stable IPF and AE-IPF based on the newly published international guidelines. Serum levels of four biomarkers at admission were measured by the enzyme-linked immunosorbent assay (ELISA). The primary endpoint was 3-month mortality. The log-rank test and logistic regression analysis were used to evaluate the effects of these biomarkers for survival in patients with AE-IPF. Cox proportional hazards analysis was performed to evaluate the prognostic values of serological biomarkers and clinical data.

**Results:**

Eighty-one patients were diagnosed with stable IPF, and 41 AE-IPF patients were enrolled in the study. Serum levels of sICAM-1 (*p* < 0.001), IL-1*β* (*p* < 0.001), MIF (*p* < 0.001), and su-PAR (*p* < 0.001) in patients with IPF were significantly increased compared to those in healthy controls. All the four biomarkers were elevated in patients with AE-IPF compared to those with stable IPF. The 3-month mortality in AE-IPF was 56.1% (23/41). Increased levels of MIF (*p* = 0.01) and IL-1*β* (>5 pg/mL, *p* = 0.033) were independent risk factors for 3-month mortality in patients with AE-IPF.

**Conclusions:**

We showed the higher serum levels of IL-1*β*, and MIF had prognostic values for 3-month mortality in AE-IPF. This study provided a clue to promote our understanding in the pathogenesis of the disease.

## 1. Introduction

Idiopathic pulmonary fibrosis (IPF) is a progressive, interstitial lung disease with unknown etiologies [1, 2]. The clinical course of IPF is highly heterogeneous. Some patients could remain relatively stable with slow decline of pulmonary function, while others may suffer sudden deterioration so called acute exacerbation of idiopathic pulmonary fibrosis (AE-IPF). AE-IPF is characterized as an acute worsening of dyspnea and newly emerging ground glass opacities (GGO) on the background of a usual interstitial pneumonia (UIP) pattern in high-resolution computed tomography (HRCT), which has poor prognosis for the median survival of approximately 3-4 months [3–5]. But the pathogenesis of AE-IPF is still unclear.

Increasing evidences indicate that acute inflammation may be responsible for the occurrence of AE-IPF rather than an abrupt fibrosis aggravation. In addition, the activation of abnormal coagulation and fibrinolysis pathways has been implicated in AE-IPF for elevated levels of plasma D-dimer and fibrin degradation product (FDP) observed in patients with AE-IPF [6]. Proinflammatory cytokines intercellular adhesion molecule 1 (sICAM-1) and interleukin 1*β* (IL-1*β*) have been reported to be associated with another severe condition, acute respiratory distress syndrome (ARDS), which shares several pathophysiological features with AE-IPF [7, 8]. Also, macrophage migration inhibitor factor (MIF) is an inflammatory and stress-regulating cytokine with chemokine-like functions that play critical roles in inflammatory diseases and atherogenesis [9, 10]. Coagulation-related cytokine soluble urokinase plasminogen activator receptor (su-PAR) is associated with higher mortality in ARDS [11]. However, prognostic values of these biomarkers in AE-IPF remain unknown.

In this study, we measured serum levels of these four cytokines in patients with IPF and age-matched healthy controls. Next, we compared their differential expressions between patients with stable IPF and AE-IPF. And then, we investigated their prognostic values for predicting 3-month mortality in patients with AE-IPF and compared them with the existing clinical data.

## 2. Materials and Methods

### 2.1. Study Subjects

From August 2017 to November 2019, 122 consecutive patients with IPF were admitted to the Department of Pulmonary and Critical Care Medicine, Nanjing Drum Tower Hospital. The diagnosis criteria of stable IPF and AE-IPF were based on the updated international guidelines and made by a multiple disciplinary team consisted of experienced respiratory physicians and radiologists. Briefly, the criteria for a diagnosis of IPF were as follows: (a) exclusion of other known causes of interstitial lung disease (ILD), (b) the presence of a UIP pattern on HRCT in patients not subjected to surgical lung biopsy, and (c) specific combinations of HRCT and surgical lung biopsy pattern in patients subjected to surgical lung biopsy. The diagnosis of AE-IPF was acute worsening or development of dyspnea typically less than one month with new emerging GGO on the background of the UIP pattern in HRCT. Other conditions leading to a clinical exacerbation such as acute heart failure and pulmonary embolism should be excluded [12].

Secondary interstitial lung fibrosis was excluded through clinical and laboratory investigations. To assess the prognostic value of coagulation dysfunction in patients with IPF, patients who had combination of other thrombotic diseases including acute-stage cerebral infarction and deep vein thrombosis or who had received anticoagulation therapy within 6 months were excluded. The stable patients have to be in a stable phase for at least 3 months before being enrolled in the study.

Baseline demographic information; clinical characteristics and lab examinations including lactate dehydrogenase (LDH), C-reactive protein (CRP), erythrocyte sedimentation rate (ESR), D-dimer, and albumin (ALB); and blood routine examination including white blood cell count and neutrophil percentage and the PaO_2_/FiO_2_ ratio were collected. Pulmonary function data at admission were available in 61 patients (50 patients with stable IPF and 11 patients with AE-IPF). Variables including forced vital capacity (FVC), FVC% predicted, diffusing capacity of the lung for carbon monoxide (DLCO), and DLCO% predicted were recorded. The Acute Physiology and Chronic Health Evaluation II (APACHE II) score was calculated within 24 hours after the patients were diagnosed with AE. The primary endpoint was 3-month mortality. Survival status was confirmed by reviewing medical documents and telephone follow-up. Survival time of AE-IPF was calculated from the diagnosis of AE to death.

Forty-five age- and-gender-matched healthy controls were from the Physical Examination Center of Nanjing Drum Tower Hospital. This study was approved by the Ethics Committee at Nanjing Drum Tower Hospital (protocol number 2016-138-01, November 15, 2016).

### 2.2. Blood Sample Collection

Peripheral blood samples (5 mL) were collected using standardized venipuncture procedures. Blood samples of patients with stable IPF were collected at the first day of admission. Blood samples of patients with AE-IPF were collected when the diagnosis of AE-IPF was confirmed. Serum samples were separated by centrifugation and stored at −80°C.

### 2.3. Measurement of Serum Biomarkers

Commercially available enzyme-linked immunosorbent assay (ELISA) kits were adopted to measure the serum levels of sICAM-1 and IL-1*β* (Lianke Corporation, Wuhan, China), MIF (Cusabio Biotechnology Corporation, Wuhan, China), and su-PAR (Ousaid Biotechnology Corporation, Changsha, China) according to the manufacturers' instructions, and every sample had a duplicate. The mean minimum detectable dose by the ELISA kits was 8.81 pg/mL.

### 2.4. Statistical Analysis

Numerical variables were expressed as mean ± SD. Differences of biomarkers among groups were examined by Mann-Whitney *U* tests. Student's *t*-test was applied to compare continuous clinical variables when they satisfied a normal distribution. Categorical variables were compared by the chi-square test. Survival of AE and stable IPF patients was evaluated using the logistic regression analysis and log-rank test. The rank correlation analysis was performed to evaluate the associations between cytokines and clinical data. Logistic regression analysis was used to filtrate important confounders regarding the prognosis of AE-IPF. If the regression coefficient of the variables was statistically significant, it will be introduced in multivariate models. Cox proportional hazards analysis was used to identify significant predictors of 3-month mortality in patients with AE-IPF. *p* values < 0.05 were considered to be significant. Data analyses were performed using SPSS statistical software version 22.0.

## 3. Results

### 3.1. Subject Characteristics

**Figure 1:**
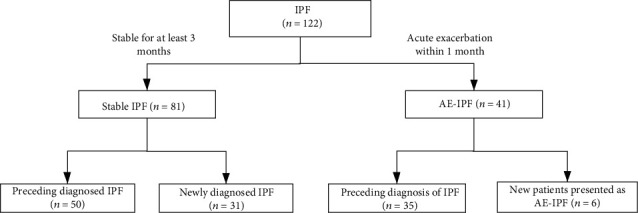
Flowchart of the patients' enrollment. Thirty-seven patients were newly diagnosed with IPF; eighty-five patients had previously been diagnosed with IPF, and 35 of them develop AE-IPF.

During the study period, 122 consecutive patients with IPF were admitted to the Department of Pulmonary and Critical Care Medicine, Nanjing Drum Tower Hospital. They were 102 males (83.6%) and 20 females (16.4%), with mean age of 65.8 ± 8.6 years old. Among them, 81 patients were diagnosed with stable IPF (65.2 ± 8.8 years old, ranging from 37 to 84) and 41 patients with AE-IPF (66.9 ± 8.2 years old, ranging from 53 to 80). In this study, 30.3% (37/122) were newly diagnosed with IPF. All AE patients were admitted because of the aggravation of dyspnea and respiratory failure, and 41.5% (17/41) of them also have suspectable pulmonary infections. At admission, 85.4% (35/41) of AE patients have a preceding diagnosis of IPF and 6 patients were newly diagnosed with AE-IPF. The flowchart is described in Figure 1. No significant differences in age, sex ratio, and smoking history were observed between the two groups. The clinical characteristics and lab examinations are summarized in Table 1. The values of ESR, CRP, LDH, ALB, WBC count, and neutrophil percentages in patients with AE-IPF were significantly higher compared to those in patients with stable IPF. The values of PaO_2_/FiO_2_, FVC% predicted, and DLCO% predicted were significantly lower in patients with AE-IPF than in those with stable IPF. An elevated serum D-dimer level was observed in patients with AE-IPF compared to those with stable IPF (*p* < 0.001).

During hospitalization, 14 patients with AE-IPF (10/41, 34.1%) received corticosteroid pulse therapy (methylprednisolone 500-1000 mg/d for 3-5 days). Three patients with AE-IPF (3/41, 7.3%) received lung transplantation. In total, there were 35 patients died in the follow-up and 25 of them were AE-IPF. The mean survival time of patients with AE-IPF was 5.4 months from diagnosis of AE-IPF, and 23 of them died within 3 months. The 3-month mortality of AE-IPF was 56.1% (23/41).

### 3.2. Serum Concentrations of sICAM-1, IL-1*β*, MIF, and su-PAR

**Figure 2:**
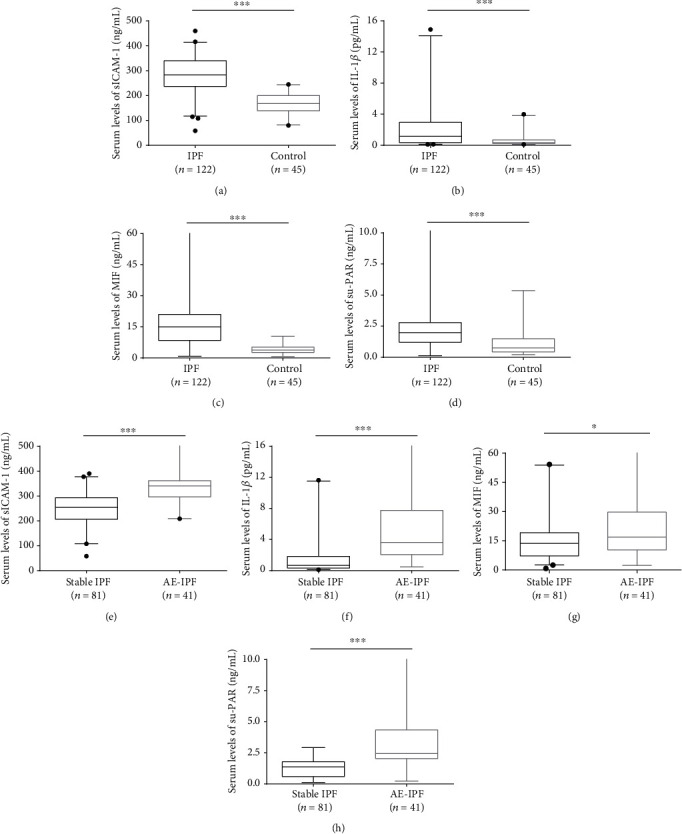
(a–d) Serum levels of sICAM-1, IL-1*β*, MIF, and su-PAR in healthy controls and all patients with IPF. (e–h) Serum levels of sICAM-1, IL-1*β*, MIF, and su-PAR in patients with stable IPF and AE-IPF. Boxes represent all the values in the groups, solid lines within the boxes show the median values, and whiskers are the 10th and 90th percentiles. AE-IPF: acute exacerbation of idiopathic pulmonary fibrosis; IPF: idiopathic pulmonary fibrosis. ^∗^*p* < 0.05 (Mann-Whitney *U* test). ^∗∗∗^*p* < 0.001 (Mann-Whitney *U* test).

Serum levels of sICAM-1 (*p* < 0.001), IL-1*β* (*p* < 0.001), MIF (*p* < 0.001), and su-PAR (*p* < 0.001) in patients with IPF were significantly higher than those in healthy controls. Serum levels of sICAM-1 (*p* < 0.001), IL-1*β* (*p* < 0.001), MIF (*p* = 0.038), and su-PAR (*p* < 0.001) in patients with AE-IPF were significantly higher than those in patients with stable IPF (Figure 2).

### 3.3. Correlations between Serum Levels of sICAM-1, IL-1*β*, MIF, and su-PAR and Other Clinical Markers

In all patients, Spearman's rank correlation coefficient revealed that the serum levels of su-PAR were correlated with D-dimer (*r*_s_^2^ = 0.379, *p* = 0.001, *n* = 122). The baseline levels of serum IL-1*β* were correlated with WBC count (*r*_s_^2^ = 0.361, *p* = 0.001, *n* = 122), CRP (*r*_s_^2^ = 0.391, *p* = 0.001, *n* = 122), LDH (*r*_s_^2^ = 0.481, *p* < 0.001, *n* = 122), and ESR (*r*_s_^2^ = 0.367, *p* = 0.002, *n* = 122). The serum levels of sICAM-1 were correlated with WBC count (*r*_s_^2^ = 0.330, *p* = 0.004, *n* = 122), not with CRP, ESR, and LDH. The serum levels of MIF were not correlated with any of these clinical parameters. However, in patients with AE-IPF, the serum levels of su-PAR were not correlated with D-dimer. In addition, the serum levels of sICAM-1, IL-1*β*, and MIF were not correlated with these inflammatory-related laboratory data. Therefore, in patients with AE-IPF, it is meaningful to analyze the impact of these biomarkers on 3-month mortality.

### 3.4. Prognostic Value of Serum IL-1*β*, MIF, and su-PAR in AE-IPF

**Figure 3:**
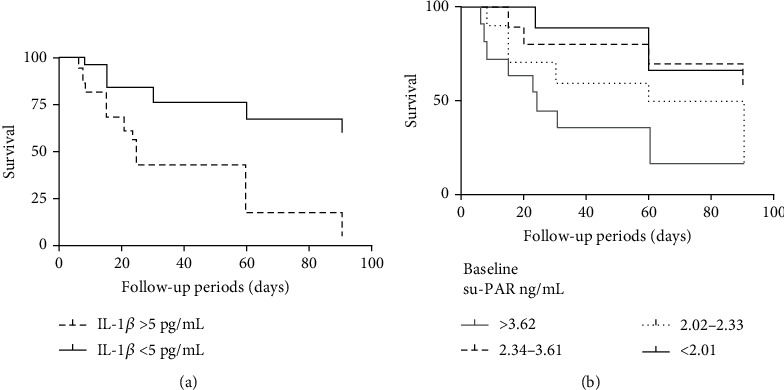
Kaplan–Meier based on serum levels of IL-1*β* (a) and su-PAR (b) estimates the 3-month mortality of AE-IPF: (a) log-rank test (*p* < 0.001) (IL‐1*β* > 5 pg/mL, IL‐1*β* < 5 pg/mL); (b) log-rank test (*p*_1_ = 0.012) (su‐PAR > 3.627 ng/mL, su‐PAR < 2.01 ng/mL).

The mean follow-up time in patients with AE-IPF was 7.6 months (range from 3 to 23 months). Logistic regression analysis revealed that higher serum levels of MIF (*p* = 0.007), su-PAR (*p* = 0.033), and IL-1*β* (*p* = 0.018) were associated with poor prognosis in patients with AE-IPF. However, higher levels of sICAM-1 were not a risk factor for survival. We performed survival analysis in patients with AE-IPF. Firstly, all these biomarkers were analyzed as continuous variables. Then, categorizations of these variables were taken into consideration when the former were not appropriate. The normal range of IL-1*β* in our center laboratory was less than 5 pg/mL. The log-rank test was performed according to the clinical cut-off levels and demonstrated that the increased levels of IL-1*β* (>5 pg/mL) were associated with higher 3-month mortality in patients with AE-IPF (*p* < 0.001) (Figure 3). The baseline su-PAR concentrations in patients with AE-IPF were 3.13 ng/mL (0.23-8.13 ng/mL). Patients were divided into four groups of approximately equal numbers according to the quartile (2.021 ng/mL, 2.345 ng/mL, and 3.627 ng/mL). The log-rank test showed that the group with the highest quartile of baseline su-PAR concentrations had higher risk for 3-month mortality when compared to the lowest quartile (*p* = 0.012) (Figure 3). Logistic regression analysis revealed that the APACHE II score, PaO_2_/FiO_2_ ratio, D-dimer, and WBC counts were more likely to be related to prognosis (*p* = 0.273, 0.075, 0.104, and 0.043, respectively).

Demographics and the existing clinical data were analyzed firstly. Univariate analysis revealed that only WBC count was associated with worse 3-month mortality. A Cox proportional hazards model revealed that increased serum levels of MIF were independently associated with worse survival after adjustment for the PaO_2_/FiO_2_ ratio, APACHE II score, and WBC count (OR = 1.001, 95% CI: 1.000-1.001, *p* = 0.01). The elevated serum levels of IL-1*β* (>5 pg/mL) were associated with worse survival after adjustment for related markers (OR = 2.548, 95% CI: 1.080-6.012, *p* = 0.033). After adjustment for demographic information (age, sex, and smoking history) in multivariate model 2, the above conclusion is appropriate (Table 2). However, the effects on 3-month mortality of higher su-PAR and D-dimer do not have statistical difference.

## 4. Discussion

The pathogenesis of AE-IPF remains incompletely understood. Previous studies reported that Asian populations may be at a higher risk of AE-IPF than Caucasian ones, due to different genetic risk factors of IPF among ethnicities [13, 14]. We showed that serum levels of sICAM-1, IL-1*β*, MIF, and su-PAR were significantly elevated in patients with IPF compared to healthy controls. All of these four biomarkers were significantly higher in patients with AE-IPF than in those with stable IPF. Cox proportional hazards analysis demonstrated that higher levels of IL-1*β* and MIF were significant risk factors for predicting 3-month mortality in patients with AE-IPF. This study provided evidences that acute inflammatory response and coagulation abnormalities may participate in the pathogenesis of AE-IPF.

The sICAM-1, a member of the immunoglobulin supergene family, is expressed on the surface of vascular endothelial cells at low levels in physiological conditions. Its soluble form can be regarded as a marker for endothelial as well as alveolar epithelial damage [15]. Previous studies showed that increased level of sICAM-1 was associated with poor prognosis in patients with acute lung injury [16, 17]. In this study, higher levels of sICAM-1 were not a risk factor for survival in patients with AE-IPF. There is further evidence that the abrupt fibrosis aggravation is not the main pathogenic mechanism of AE-IPF. As an inflammatory mediator, IL-1*β* was mostly secreted by activated monocytes and macrophages. The activation of WNT/*β*-catenin in the development of pulmonary fibrosis may lead to the alveolar epithelium being a relevant source of IL-1*β* [18]. Previous studies revealed that IL-1*β* could reflect the acute inflammatory response in some pulmonary diseases such as asthma and ARDS [19, 20]. In this study, we found that serum levels of IL-1*β* increased only in the AE-IPF group, indicating that the inflammatory response in AE-IPF is more intensive than that in stable IPF.

MIF is known as a pleiotropic inflammatory mediator in the innate and adaptive immune responses. It presents in most immune cells, such as T cells, macrophages, and monocytes. Soluble MIF could promote neutrophil accumulation via binding with the CD74 receptor of macrophages in pulmonary inflammation [21–23]. Serum levels of MIF were increased in patients with IPF combined with pulmonary hypertension, and MIF levels in lung tissues and bronchoalveolar lavage fluids (BALF) were significantly increased in the murine model of bleomycin-induced pulmonary fibrosis [24]. Li et al. reported that MIF was elevated in patients with acute kidney injury (AKI) and blocking MIF could alleviate the AKI in model mice [25]. In our study, serum levels of MIF were elevated in patients with AE-IPF and were associated with poor survival in them. Furthermore, values of inflammation indexes such as ESR, CRP, LDH, WBC count, and neutrophil percentage in patients with AE-IPF were significantly higher compared to those in patients with stable IPF. Together, our findings implicated that acute inflammatory responses played an important role in AE-IPF.

Accumulating evidences suggested extensive cross-talks between the inflammatory response and abnormal coagulation process. During the acute inflammation process, proinflammatory cytokines and endotoxin activated the coagulation pathway [26]. In pathological conditions, disturbed expression of a tissue factor caused by endothelial integrity destruction played an essential role in the activation of the clotting cascade. Also, the activation of coagulation could enhance inflammatory responses by increasing vascular permeability, producing proinflammatory mediators and recruiting more neutrophils [27]. As one of the members of the newly identified plasminogen activator (PA) family, urokinase plasminogen activator receptor (uPAR) is active in the inflammation and coagulation pathway [28]. The su-PAR was regarded as a biomarker for disease severity in ARDS [11]. Our study firstly showed that higher levels of su-PAR are associated with poor prognosis of AE-IPF. However, this study failed to prove that the higher levels of su-PAR were associated with 3-month mortality. Kondoh et al. reported that the thrombomodulin alfa did not improve the 3-month survival proportion in patients with AE-IPF [29]. Therefore, inflammatory factor storms may be more responsible for the short-term death of AE-IPF.

Corticosteroids were a wide spread treatment option in AE-IPF [30]. But the controversy about the usage in AE-IPF is still heat. Farrand et al. reported that corticosteroid was not associated with improved outcomes in acute exacerbation of IPF [31]. A randomized controlled trial (RCT) from Japan showed that acute exacerbation was significantly more frequent in the placebo group compared to the pirfenidone treatment group [32]. More recently, INPULSIS trials suggested that nintedanib may reduce the risk of developing an acute exacerbation [33]. Debates still existed about whether anticoagulant therapy should be given to patients with AE-IPF. A previous study showed that mortality in patients with AE-IPF who received anticoagulation therapy was lower than that in patients who did not [34]. A post hoc analysis suggested that the anticoagulants (most were taking the vitamin K antagonist warfarin) had unfavorable effects in IPF patients [35]. Future well-designed RCT studies are needed to investigate the necessity of anticoagulant therapy in AE-IPF.

This study has several limitations. First, this study was conducted in a single center, so selection bias was inevitable and the prognostic values of these biomarkers should be validated in further study including multiple centers. Second, the number of confounders was restricted due to the small number of the outcome (death). In addition, only a handful of AE-IPF patients in our study underwent bronchoalveolar lavage, which limited the possibility to compare the levels of these biomarkers in BALF and serum.

In conclusion, increased levels of sICAM-1, IL-1*β*, MIF, and su-PAR were observed in AE-IPF. The increased levels of IL-1*β* and MIF were risk factors for 3-month mortality in patients with AE-IPF. Our study provided evidences that acute inflammatory response and coagulation abnormalities may participate in the pathogenesis of AE-IPF.

## Figures and Tables

**Table 1:** Comparison of clinical characteristics in patients with stable IPF and AE-IPF.

Clinical characteristics	Stable IPF (*n* = 81)	AE-IPF (*n* = 41)	*p* value (stable vs. AE)
Age (years)	65.2 ± 8.8	66.9 ± 8.2	0.257
Sex M/F (*n*)	70/11	32/9	0.240
Smoking history	32.7% (35)	51.2% (21)	0.404
FVC% predicted	71.9 ± 16.2 (*n* = 65)	55.3 ± 16.3 (*n* = 11)	0.003^∗^
DLCO% predicted	57.4 ± 20.59 (*n* = 65)	28.0 ± 11.0 (*n* = 11)	<0.001^∗^
PaO_2_/FiO_2_	339 ± 119.7	181.5 ± 86.4	<0.001^∗^
WBC (10^3^/*μ*L)	6.7 ± 1.7	10.5 ± 4.4	<0.001^∗^
Neutrophils (%)	57.9 ± 9.5	81.8 ± 13.4	<0.001^∗^
ESR	22.2 ± 17.6	46.8 ± 26.3	<0.001^∗^
LDH (U/L)	234.4 ± 52.3	489.6 ± 370.9	<0.001^∗^
CRP (mg/mL)	7.4 ± 12.3	47.7 ± 60.5	<0.001^∗^
ALB (g/L)	38.6 ± 2.0	33.8 ± 4.1	<0.001^∗^
Platelet (×10^4^/*μ*L)	192.5 ± 61.0	221.8 ± 73.7	0.061
D-dimer (mg/mL)	0.5 ± 0.4	4.4 ± 8.4	<0.001^∗^
Survival rate	83.9%	39.0%	<0.001^∗^
3-month mortality	—	56.1%	

Values are mean ± SD unless stated otherwise. WBC: white blood cell; LDH: lactate dehydrogenase; CRP: C-reactive protein; ESR: erythrocyte sedimentation rate; ALB: albumin; DLCO: diffusing capacity of the lung for carbon monoxide; FVC: forced vital capacity. ^∗^*p* < 0.05 (Mann-Whitney *U* test). The mean follow-up time of patients with stable IPF was 15.6 months, and the mean follow-up time of patients with AE-IPF was 7.6 months.

**Table 2:** Multivariate analysis of variables associated with 3-month mortality in patients with AE-IPF.

Variables	Univariate model			Multivariate model		
	Hazard ratio	95% CI	*p* value	Hazard ratio	95% CI	*p* value
Age (years)	1.003	0.954-1.054	0.906			
Sex, male	1.332	0.499-3.554	0.567			
Smoking history	1.528	0.692-3.372	0.294			
APACHE II score	1.076	0.982-1.179	0.112			
FVC% predicted	0.970	0.905-1.039	0.376			
DLCO% predicted	0.974	0.889-1.086	0.578			
PaO_2_/FiO_2_ ratio	0.996	0.991-1.001	0.084			
LDH (U/L)	1.001	1.000-1.001	0.264			
WBC (10^3^/*μ*L)	1.091	1.007-1.182	0.032^∗^			
D-dimer (mg/mL)	1.026	0.990-1.064	0.153			
Model 1						
IL-1*β*, continuous	1.078	0.997-1.165	0.060	1.094	1.003-1.194	0.044
IL‐1*β* > 5 pg/mL	2.473	1.107-5.526	0.032^∗^	2.538	1.080 -6.012	0.033^∗^
MIF, continuous	1.001	1.000-1.001	<0.001^∗^	1.001	1.000-1.001	0.01^∗^
su-PAR, continuous	1.163	0.990-1.366	0.063	1.151	0.977-1.356	0.093
Model 2						
IL-1*β*, continuous	1.078	0.997-1.165	0.060	1.069	0.974-1.172	0.16
IL‐1*β* > 5 pg/mL	2.473	1.107-5.526	0.032^∗^	2.424	0.914-6.432	0.045^∗^
MIF, continuous	1.001	1.000-1.001	<0.001^∗^	1.0001	1.000-1.001	0.001^∗^
su-PAR, continuous	1.163	0.990-1.366	0.063	1.200	0.990-1.456	0.064

A Cox proportional hazards model revealed that higher serum levels of IL-1*β* (>5 pg/mL) and MIF were independent predictors of the mortality of AE after adjustment for the PaO_2_/FiO_2_ ratio, APACHE II score, and WBC in model 1. In addition, when IL-1*β* was a continuous variable, multivariate model 1 was not statistically significant. Higher serum levels of IL-1*β* and MIF were independent predictors of the mortality of AE after adjustment for age, sex ratio, and smoking history in model 2. In multiple Cox regression of su-PAR, D-dimer was induced additionally. ^∗^*p* < 0.05.

## Data Availability

Clinical and experimental data are available when needed.

## Abbreviations

IPF: Idiopathic pulmonary fibrosis
AE-IPF: Acute exacerbation of idiopathic pulmonary fibrosis
ARDS: Acute respiratory distress syndrome
AKI: Acute kidney injury
APACHEII score: Acute physiology and chronic health evaluation II score
GGO: Ground glass opacity
UIP: Usual interstitial pneumonia
sICAM-1: Intercellular adhesion molecule 1
MIF: Macrophage migration inhibitor factor
IL-1*β*: Interleukin 1*β*
su-PAR: Soluble urokinase plasminogen activator receptor
WBC: White blood cell
LDH: Lactate dehydrogenase
CRP: C-reactive protein
FDP: Fibrin degradation product
ESR: Erythrocyte sedimentation rate
ALB: Albumin
FVC: Forced vital capacity
DLCO: Diffusing capacity of the lung for carbon monoxide
HRCT: High-resolution computed tomography
ELISA: Enzyme-linked immunosorbent assay
CI: Confidence interval
HR: Hazard ratio
BALF: Bronchoalveolar lavage fluids
RCT: randomized controlled trial.
